# Skin-inspired highly stretchable and conformable matrix networks for multifunctional sensing

**DOI:** 10.1038/s41467-017-02685-9

**Published:** 2018-01-16

**Authors:** Qilin Hua, Junlu Sun, Haitao Liu, Rongrong Bao, Ruomeng Yu, Junyi Zhai, Caofeng Pan, Zhong Lin Wang

**Affiliations:** 1 CAS Center for Excellence in Nanoscience, Beijing Institute of Nanoenergy and Nanosystems, Chinese Academy of Sciences, Beijing, 100083 China; 2 School of Nanoscience and Technology, University of Chinese Academy of Sciences, Beijing, 100049 China; 3 Institute of Microelectronics, Tsinghua University, Beijing, 100084 China; 4 School of Materials Science and Engineering, Georgia Institute of Technology, Atlanta, GA 30332-0245 USA

## Abstract

Mechanosensation electronics (or Electronic skin, e-skin) consists of mechanically flexible and stretchable sensor networks that can detect and quantify various stimuli to mimic the human somatosensory system, with the sensations of touch, heat/cold, and pain in skin through various sensory receptors and neural pathways. Here we present a skin-inspired highly stretchable and conformable matrix network (SCMN) that successfully expands the e-skin sensing functionality including but not limited to temperature, in-plane strain, humidity, light, magnetic field, pressure, and proximity. The actualized specific expandable sensor units integrated on a structured polyimide network, potentially in three-dimensional (3D) integration scheme, can also fulfill simultaneous multi-stimulus sensing and achieve an adjustable sensing range and large-area expandability. We further construct a personalized intelligent prosthesis and demonstrate its use in real-time spatial pressure mapping and temperature estimation. Looking forward, this SCMN has broader applications in humanoid robotics, new prosthetics, human–machine interfaces, and health-monitoring technologies.

## Abstract (web summary)

Electronic skins have been developed to emulate human sensory systems, but simultaneous detection of multiple stimuli remains a big challenge due to coupling of electronic signals. Here, Hua et al. overcome this problem in a stretchable and conformable matrix network integrated with seven different modes.

## Introduction

The human somatosensory system is a complex network that converts environmental stimuli into electrical impulses through various sensory receptors (mechanoreceptors, thermoreceptors, nociceptors, etc.) and transmits these signals via neural pathways, enabling the sensations of touch, heat/cold, and punching invasion. Consisting of mechanically flexible and stretchable sensor networks, mechanosensation electronics (electronic skin, e-skin)^1–6^ has been developed to mimic the human somatosensory system by detecting and quantifying various stimuli in the ambient environment and have attracted tremendous attention for their revolutionary applications in robotics^7,8^, prosthetics^4,9,10^, and health-monitoring technologies^3,11,12^. E-skin, which is capable of sensing different stimuli, is likely to boost emergence of the Internet of ‘actions’ (IoA), as we suppose, which would be a new era of health care, medical science, and robotics. The IoA would ascribe a world where billions of objects tightly integrated with sensors, processors, and actuators to sense stimuli and do actions interactively and adaptively; could naturally allow people to interact and communicate with the surroundings, including physical objects and external stimuli; and do some actions in response after computations^13^.

Mechanosensation electronics is supposed to be the core part of IoA^2,11,14–16^, and multi-functionalities are of essential importance in developing smart and interactive flexible/stretchable electronics^2,15–17^. Indeed, the ability to sense multiple stimuli is an ultimate goal for e-skin systems^3–5^. However, previous reports have mainly focused on single or dual sensory capabilities. Many emerging sensors, including pressure^18–21^, strain^22–28^, and temperature^29,30^, are underway to achieve excellent performance. Strain and temperature sensing were demonstrated by whisker arrays^22^ and thin film sensors^31^. Pressure sensing coupled with temperature (e.g., nanocomposites^32^, organic transistors^33^, thermoelectric^34^, or ferroelectric^35^ materials), strain (e.g., carbon nanotubes^36^, gold thin films^26^, or liquid conductors^37^ in silicone rubber, interlocked micro/nanostructures^25,38,39^, elastomer-based integrated tactile sensors^10^, energy-harvesting e-skin^40^), or proximity^41,42^ were also illustrated. Epidermal electronics^11,43^, piezoelectric sensors^44^, and micro-hairy sensors^12^ showed cutaneous and physiological monitoring. Si-nanoribbon strain, pressure, and temperature sensor arrays associated with humidity sensors and heaters were reported in a prosthetic skin^9^, but it did not show evidence of the sensory inputs recorded simultaneously. Surely, highly sensitive detections for different stimuli are not easy to implement due to decoupling interference of multiple signal^34^. Thus a simultaneous detection and high selectivity of multi-complex stimuli from the ambient environment remains a challenge, which puts high demands on a high-density sensor array^4^. Additionally, mechanoreceptor density varies widely for different areas of skin for a human, such as between fingertips (241 cm^−2^) and palms (58 cm^−2^)^45^. However, few reported e-skins achieved adjustable sensing range and area expansion^46–48^ in analogy of mechanoreceptors of the human skin.

Here we present a skin-inspired highly stretchable and conformable matrix network (SCMN) as a multi-sensory e-skin that is capable of detecting temperature, in-plane strain, relative humidity (RH), ultraviolet (UV) light, magnetic field, pressure, and proximity provides to realize simultaneous multi-stimulus sensing and exhibits an adjustable sensing range and large-area expandability, as well as potentially suitable for high-density three-dimensional (3D) integration scheme. More impressively, we likewise construct a personalized intelligent prosthetic hand for touch/temperature sensing that not only contour pressure distribution on fingers but also estimate temperatures of grasping objects simultaneously. This is a milestone using mechanosensation electronics toward IoA.

## Results

### Skin-inspired SCMN for multifunctional sensing

**Fig. 1:**
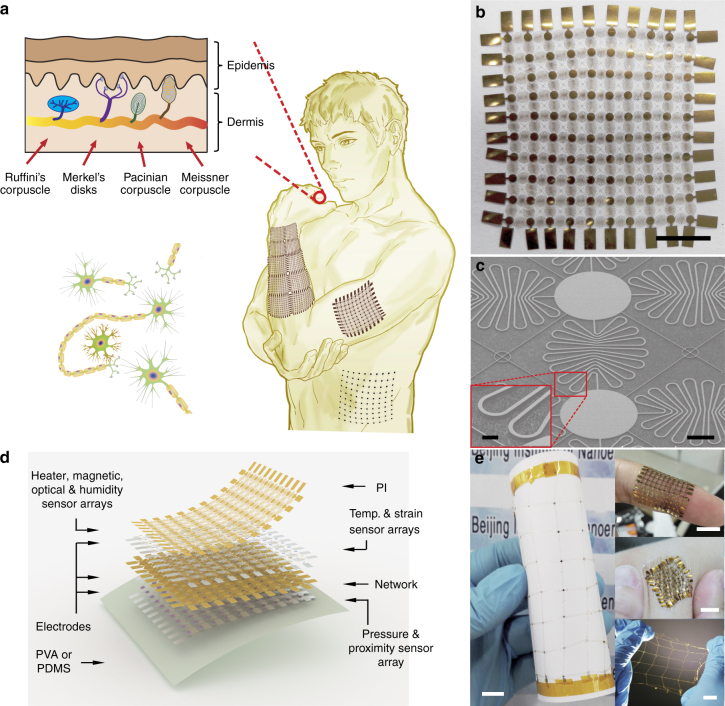
Skin-inspired highly stretchable and conformable matrix networks. **a** Schematic illustration of SCMNs conforming to the surface of a human arm and an expanded network (expansion: 200%) conforming to the surface of a human abdomen (right); the tree branch-like connections of neurons (left bottom); the sensory receptors of the glabrous skin (left top). **b** Optical image of the fabricated polyimide network (10 × 10 array, scale bar: 5 mm). **c** Tilted SEM image of the polyimide network (scale bar: 500 μm); the inset is a higher resolution SEM image of a meandering interconnect (scale bar: 50 μm). **d** Schematic layout of an SCMN—an integrated sensor array with eight functions. (temp.: temperature). **e** Image of an SCMN attached to a sheet of paper, demonstrating ultrahigh flexibility and stretchability. The inset (upper) shows an SCMN conforming to a human finger, (middle) an SCMN attached to human skin with compression, and (bottom) an SCMN stretched by a human hand (scale bar: 1 cm)

Figure 1a schematically illustrates the human somatosensory system in the skin, which consists of neural networks that receive and transmit touch, heat/cold, and pain signals from the external environment. Moreover, various mechanoreceptors and thermoreceptors distributed in the epidermal and dermal layers enable the spatiotemporal recognition of the magnitude and location of touch and temperature stimuli^49^. As inspired by the human skin with this complex somatosensory system, we designed and fabricated a SCMN composed of 100 sensory nodes connected by meandering wires to achieve multifunctional sensing performance using stretchable and expandable structures (Fig. 1b), with corresponding tilted SEM images shown in Fig. 1c and its inset, respectively. The multilayered design layout and the corresponding fabrication process presented in Fig. 1d and Supplementary Fig. 1, respectively, show that six different types of sensor units are responsible for the different categories of sensing performance. Such design makes the device capable of detecting temperature, RH, UV light, magnetic field, in-plane strain, and pressure/proximity stimuli from the environment simultaneously. Furthermore, the SCMN shows a very good flexibility and stretchability due to the application of meandering interconnects, which are demonstrated in Fig. 1e.

### SCMN stretchability and expandability

The meandering interconnects are critical for allowing the stable and uniform expansion of the network to several orders of magnitude larger than the original area and for the positioning of the nodes in predefined locations.

**Fig. 2:**
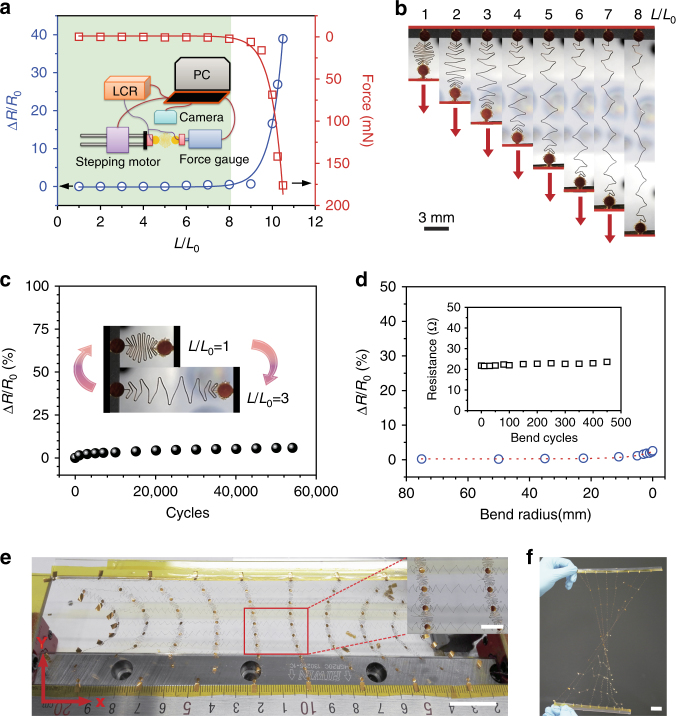
Mechanical and electrical testing of the meandering interconnects. **a** Relative resistance and tensile force change with the extension of the meandering interconnects (50-μm-wide, 50-μm-thick polyimide wire coated with Ag thin films); the green section shows a perfectly stable performance region, and the inset depicts a schematic of the customized stretch-testing platform. **b** Optical images of a meandering wire stretched from an *L*/*L*_0_ of 1 to 8 (scale bar: 3 mm). **c** Durability testing at 300% expansion, with an inset showing the stretching at *L*/*L*_0_ = 1 and *L*/*L*_0_ = 3. **d** Relative resistance change vs. bend radius, with an inset showing bend durability at a bend radius of 150 μm. **e** Optical image of an expanding SCMN (scale bar: 2 cm), with an inset depicting an enlarged view of the red rectangle (scale bar: 5 mm). The network is made of 100 nodes (1.6 mm in diameter) connected by 50-μm-wide, 25-μm-thick polyimide wires coated with Au thin films. **f** Optical image of a twisted SCMN (scale bar: 2 cm)

The stretching test is performed on a one-dimensional meandering interconnect, utilizing a customized stretch-testing platform (inset of Fig. 2a), and a detailed stretching procedure of a meandering wire can also be seen in Supplementary Movie 1. When the meandering wires are stretched from their original length (*L*/*L*_0_ = 1) to 800% expansion (*L*/*L*_0_ = 8), the extension of the wires presents a linear increase (shown in Fig. 2b), and the tensile force and resistance of the meandering wires show no obvious changes, which exhibits a superior stretchable and expandable capability. Upon mechanical deformation for over the designed ratio (*L*/*L*_0_ = 8), both the force and the resistance change exponentially with the strain, as shown in Fig. 2a. The meandering wires show a very good stability after 54,000 cycles at 300% expansion in durability tests (Fig. 2c). In addition, the relative resistance shows only a little increase (≤ 2.6%) with the reduction of bend radii; in particular, the meandering wires can be curved at a bend radius of 150 μm with good durability over 450 cycles (Fig. 2d).

By designing a matrix array of nodes connected to meandering wires, a network containing 100 nodes is successfully fabricated and placed on the customized stretch-testing platform (Supplementary Fig. 2a) to evaluate its stretchable and expandable performances. The polyimide (PI) network can be easily stretched and expanded along the desired directions (Fig. 2e and Supplementary Fig. 2b–i; Supplementary Movie 2), which achieves sensing area increase (Supplementary Fig. 2; Supplementary Movie 3), predefined sensory node location (Fig. 2f, Supplementary Figs. 2b and 3), and complex-shape object conform (Supplementary Fig. 3). These characteristics demonstrate the feasibility of SCMNs with adjustable sensing range and large-area expandability.

### Temperature sensing

**Fig. 3:**
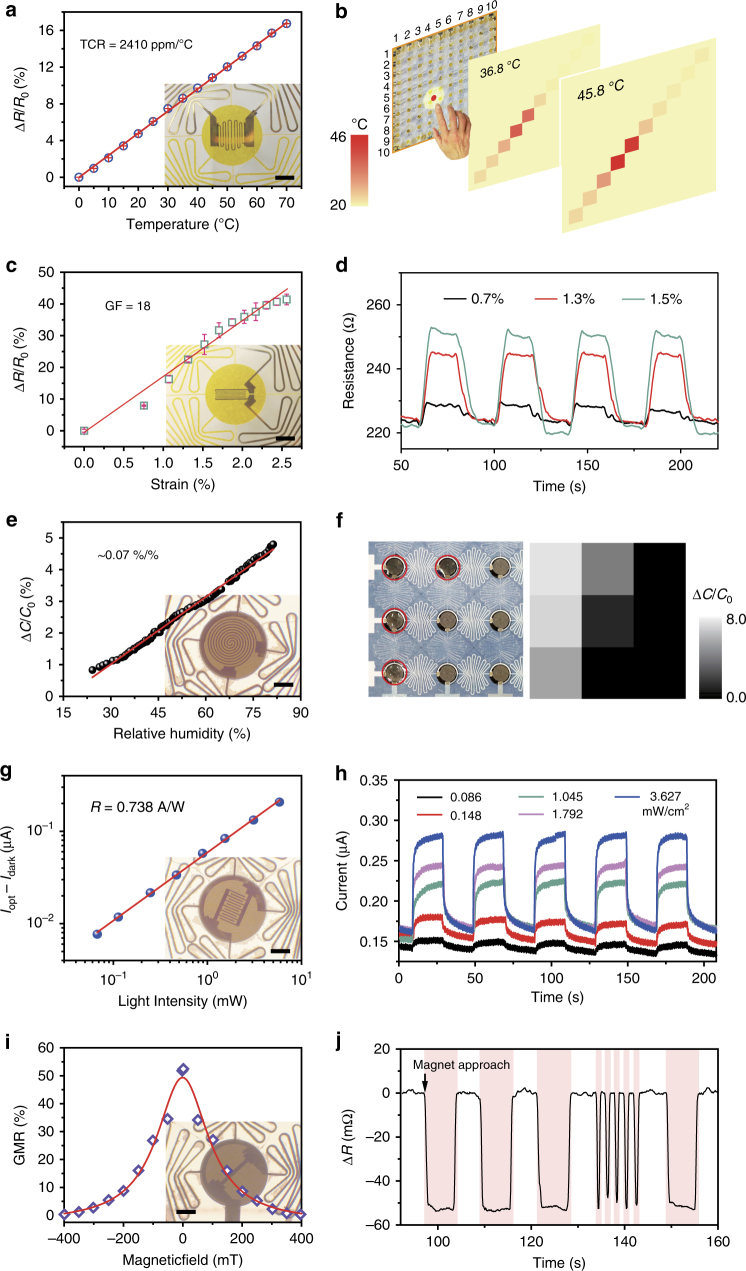
Multifunctional sensing performances. **a**, **b** Temperature sensing. **a** The relative resistance of the temperature sensor changes linearly with temperature; the inset depicts a Pt thin film temperature sensor fabricated on an SCMN. **b** Spatial temperature distribution recognition for temperatures close to 36.8 °C and 45.8 °C after placing a heat source above an SCMN. **c**, **d** In-plane strain sensing. **c** The relative resistance change increases with increasing applied strain; the inset shows a Constantan alloy thin film in-plane strain sensor mounted on an SCMN. (Error bars represent the fluctuation between normalized resistance and applied strain at each point.) **d** The temporal resistance changes for different curvatures of the strain sensor under cyclical bending (Strain: 0.7%, 1.3% and 1.5%). **e**, **f** Humidity sensing. **e** The change in capacitance is linear with increasing relative humidity (RH); the inset is an image of a humidity sensor on an SCMN. **f** Image of water droplets covering a partial area of a humidity senor array (left) and a corresponding map of the regional capacitance change (right). The red circles indicate where the droplets are placed. **g**, **h** Light sensing. **g** The photocurrent generated by UV light (355 nm) increases linearly with the light intensity; inset is an image of an optical sensor. **h** Repeatable UV response under different illumination intensities at a 5 V bias. **i**, **j** Magnetic sensing. **i** The GMR characteristics of a Co/Cu magnetic sensor; inset is an image of a magnetic sensor on an SCMN. **j** The recorded resistance changes when a magnet cyclically approaches (pink) and withdraws (white) from the sensor. Inset scale bars: 500 μm

Resistive metals, platinum (Pt) and constantan alloy (45% Ni, 55% Cu), are most commonly used as resistance temperature detectors (RTDs) and strain gauges. Pt has a higher temperature but lower strain sensitivity when compared with constantan alloy, as shown in Supplementary Figs. 4a, b. A Pt thin film is deposited on the patterned nodes of the SCMN to act as a RTD, as shown in the inset of Fig. 3a. The temperature coefficient of resistance (TCR) is a key metric for evaluating the thermal response of temperature sensors and is defined as ${\mathrm{TCR}} = \frac{1}{{R_0}} \cdot \frac{{\Delta R}}{{\Delta T}}$, where *R*_0_ is the original resistance value of the Pt thin film at 0 °C and Δ*R* is the resistance change corresponding to the temperature change Δ*T*. Fig. 3a strikingly demonstrates that the relative resistance of the temperature sensor changes linearly as the temperature increases from 0 to 70 °C, and the obtained TCR of Pt is 2410 ppm/°C, which is comparable with the TCR value of commercial products (3850 ppm/°C). Moreover, the spatial temperature distribution can be easily recognized with temperature sensors diagonally on the SCMN (Supplementary Fig. 4c), and imaging temperatures close to 36.8 °C and 45.8 °C can be differentiated clearly, as shown in Fig. 3b. Additionally, resistance responses of the temperature sensor under different stimuli (e.g., pressure, UV light, magnetic field, and RH) are presented in Supplementary Fig. 5, which surely shows a favorable selectivity in temperature for the SCMN.

### In-plane strain sensing

Constantan alloy is used in our experiment as an in-plane strain gauge due to its high strain sensitivity and low TCR (Supplementary Figs. 4a, b). The gauge factor (GF) is defined as ${\mathrm{GF}} = \frac{{\Delta R/R_0}}{\varepsilon }$, where Δ*R* is the change in resistance caused by strain *ε*, and *R*_0_ is the resistance of the undeformed gauge. As shown in Fig. 3c, the relative change of resistance is linearly correlated with the applied strain, leading to a high GF of 18. The high GF may be mechanically induced using prebent samples with various radii of curvature^50^; the detailed strain calculation method is shown in Supplementary Fig. 6a and Supplementary Note 1. In addition, the temporal resistance changes for different curvatures of the strain sensor under cyclical bending (Strain: 0.7, 1.3, and 1.5%) reveal its favorable durability for sensing different strains (presented in Fig. 3d). Our device exhibits good in-plane strain discrimination to the other external stimuli, such as RH (Supplementary Fig. 6b), magnetic field (Supplementary Fig. 6c), pressure (Supplementary Fig. 6d), and UV light (Supplementary Fig. 6e), which have very small impacts on in-plane strain sensing, as shown in Supplementary Fig. 6. Upon attachment of the strain sensor to a human finger, the bending finger manners can be recorded by the SCMN, as shown in Supplementary Fig. 6f, indicating the potential for applications in health-monitoring systems, medical diagnostic instruments, and encrypted information transmissions.

### Humidity sensing

Human skin contains no specific receptors for humidity sensing and is instead able to sense changes in humidity via mechanoreceptors and thermoreceptors^51^. In our device, a fabricated capacitor-based sensor, as shown in the inset of Fig. 3e, is used to sense ambient RH. The absorption of water molecules changes the permittivity of the PI and thus the capacitance of the humidity sensor, yielding a linear correlation between relative changes in capacitance and RH, which results in a slope of 0.07 (Fig. 3e). The fast response times of the humidity sensor for absorption (1.5 s) and desorption (50.6 s) are presented in Supplementary Fig. 7a. A good RH selectivity in multi-stimulus sensing of SCMN is presented as well, external stimuli, such as pressure loads (Supplementary Fig. 7b), temperature changes (Supplementary Fig. 7c), magnetic fields (Supplementary Fig. 7d), and in-plane strain (Supplementary Fig. 7e), have negligible effects on humidity sensing. Additionally, the capacitances of RH sensing do not show any noticeable changes although capacitance signals exhibit larger disturbances when UV light (3 mW cm^−2^) turns on (Supplementary Figs. 7f). Spatial humidity mapping clearly discriminates areas of the humidity sensor arrays that are only partially covered by water droplets (Fig. 3f), demonstrating the potential for use in detecting spatial differences in RH.

### Light sensing

Sensing ambient light, including UV, visible and infrared (IR) light, is another important function of the artificial human somatosensory system. Humans can neither see nor differentiate the illumination intensity of UV or IR light, neither with eyes nor skins. Overexposure to UV or IR light can be harmful to the human body, hence it is necessary to achieve UV or IR light e-skin detecting. Additionally, the inclusion of optical sensors in e-skin would broaden the vision sensing range for humans. Here we take UV sensor based on ZnO as an example, while IR sensor, which can be achieved by depositing the corresponding sensitive material on the interdigital electrodes, is not demonstrated in this work yet. ZnO-based metal–semiconductor–metal sensors were integrated into the network, because ZnO is regarded as one of the most promising candidates for UV photodetection^52^. As shown in Supplementary Fig. 8a, a remarkable increase is observed in the current induced by 355 nm UV illumination (286.5 mW cm^−2^) at a 5 V bias, with a rapid response time at the rising edge of 41 ms (Supplementary Fig. 8b). Moreover, photocurrent induced by UV light is linear with respect to illumination intensity, resulting in a fast photo-responsivity *R* of 0.738 A/W, as shown in Fig. 3g. The good repeatability of UV sensors is investigated under various UV illumination intensities, as presented in Fig. 3h. External disturbances such as magnetic fields (Supplementary Fig. 8c), RH (Supplementary Fig. 8d), strain (Supplementary Fig. 8f), and pressure (Supplementary Fig. 8g) have negligible effects on UV light sensing. Normalized photocurrents exhibit a small growth due to the excited electron concentration of the conduction band in ZnO increasing as temperature (Supplementary Fig. 8e) but which would have a small impact on UV discrimination from other stimuli. Moreover, it is feasible to replace ZnO thin film with other materials, such as CdS for visible light and Ge/Si for IR light, implying the potential to expand light sensing by SCMNs to a wider spectral range.

### Magnetic field sensing

Highly sensitive, giant magnetoresistive (GMR)-sensing elements composed of Co/Cu multilayers are deposited on the sensory nodes (inset of Fig. 3i), adding a “sixth sense”^53^ to the SCMN for the presence of static or dynamic magnetic field detection (magnetoreception). The GMR ratio ${\mathrm{GMR}}\left( {H_{\rm ext}} \right) = \left[ {R\left( {H_{\rm ext}} \right) - R_{\rm sat}} \right]/R_{\rm sat}$, is defined as the magnetic field dependence of changes in sensor resistance $R\left( {H_{\rm ext}} \right)$ and normalized to the resistance value when the sample is magnetically saturated $R_{\rm sat}$^53^. We conduct a GMR characteristic measurement of as-fabricated [Co/Cu]_50_ multilayers at room temperature. A high GMR ratio of 50% is obtained (presented in Fig. 3i), which is a typical value for Co/Cu multilayers^53,54^. Then a permanent magnet is used to approach or withdraw from the sensor equipped with [Co/Cu]_10_ multilayers alternatively. The resistance changes with respect to the intensity of the magnetic field are derived by varying the distance between the sensor and the magnetic field, as shown in Fig. 3j and Supplementary Fig. 9a. Owing to the GMR effect, the resistance is observed to drop as the magnet is moving forward, while resistance recovers to the original value as the magnet is moving backward. Furthermore, when varying each other external stimulus (e.g., temperature, RH, UV light, pressure or in-plane strain, as shown in Supplementary Figs. 9b-f), changes in resistance all present little shifts by altering the same distance between the magnet and the sensor. And the resistance variations were recorded during the application of three different strains (0.85, 1.2, and 1.47%) independently and in conjunction with an approaching magnet (pink), as shown in Supplementary Fig. 9g, indicating that magnetic field sensing can be differentiated easily when applied strains. In short, this magnetoreceptive e-skin enables magnetic field proximity detection, navigation, and touchless control and essentially intensifies the sensing range of the e-skin.

**Fig. 4:**
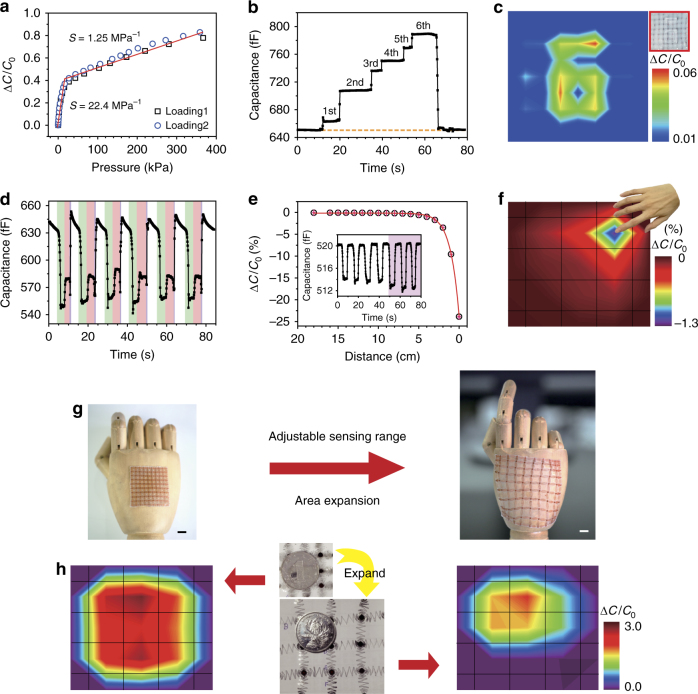
Pressure and proximity sensing performances and area expansion. **a** Capacitance response to various pressures for two consecutive load tests. **b** Real-time small-pressure recording during the sequential application of six water droplets to the sensor; the corresponding pressure is 7.3 ± 1.2 Pa. **c** Pressure mapping of a number “6” stamped on the pressure sensor array with a pressure of 8 kPa. **d** Capacitance changes from a finger cyclically approaching the device (green, from a distance of approximately 1 cm), pressing it (pink) and moving away (white). **e** The relative capacitance change reduces with the decreasing distance of an approaching finger. The inset shows the capacitance difference as a finger repeatedly approaches the device. **f** Proximity detection of a hand and identification of its position. **g** Schematic illustration of an SCMN as an artificial electronic skin on a hand, showing sensing adjustability and expandability (scale bar: 1 cm). **h** Pressure mapping before and after the 300% expansion of an SCMN; the position of the pressure load is also identified after expansion

### Pressure and proximity sensing

Dual-mode capacitor-based sensors can provide both pressure and proximity sensing capabilities for e-skin, extending the tactile sensing functionality in contact and non-contact modes^24,41,42^. The pressure and proximity sensor array is fabricated on the other side of the PI substrate, which is composed of an Ecoflex dielectric layer sandwiched between Ag thin film electrode layers and exhibits comparable performance in both pressure detection and object proximity sensing.

Pressures arouse an increase in capacitance of the sensor as a result of the reduced distance between the two Ag electrodes, regardless of RH, UV light, magnetic field, temperature, or in-plane strain (Supplementary Figs. 10a-e). Pressure sensing performance is evaluated based on pressure sensitivity, which is defined as ${\mathrm{\it S}} = \frac{{\Delta C/C_0}}{{\Delta P}}$, where Δ*C* is the capacitance change, *C*_0_ is the original capacitance of the device, and Δ*P* is the change in the applied pressure. The capacitance responses to various pressures for two consecutive loading tests is shown in Fig. 4a. Under pressures <16 kPa, the sensitivity of the fabricated pressure sensor is 22.4 MPa^−1^, which is larger than previously reported values^24,36,55^. Under pressures ranging from 16 to 360 kPa, the sensitivity of the pressure sensor is 1.25 MPa^−1^, which is comparable to the record values achieved by our previously reported pressure sensor (1.45 MPa^−1^ for pressures up to 100 kPa)^24^ and by Ag nanowire pressure sensors (1.62 MPa^−1^ for pressures up to 500 kPa)^55^. In addition, pressure as low as 7.3 ± 1.2 Pa is detected and recorded in real time by the sequential application of six water droplets to the sensor, as shown in Fig. 4b. The capacitance also returns to its original value after removal of the droplets, clearly indicating the ability to detect small pressures with superior stability. Furthermore, pressure distribution mapping of the 2D surface is also demonstrated by applying a stamp in shape of a “6” to the sensor array, as shown in Fig. 4c.

Figure 4d shows capacitance changes corresponding to a human finger approaching the device (green), gently pressing it (pink), and moving away (white). These results indicate that the pressure (contact) and proximity (non-contact) sensing modes coexist, and the detailed capacitance responses of the two modes are presented in Supplementary Fig. 10f. Capacitance changes are determined with a finger repeatedly approaching the device (green, from a distance of approximately 1 cm), pressing it (pink), and then moving away (white). Finger proximity leads to a decrease in capacitance, whereas physical contact induces an increase in capacitance by reducing the distance between the two electrodes. The capacitance changes with disturbances in the fringe electric field in either contact or non-contact mode, and Supplementary Fig. 10g illustrates the capacitive working mechanism.

Proximity signals from the SCMN change with respect to the distance of an approaching finger, and the capacitance drops sharply when the distance falls below 2 cm, as shown in Fig. 4e. Furthermore, tests comparing the capacitance-reduction signals of various materials, including copper, aluminum, PVC, glass, wood, ceramic, and a human hand^41^, indicate that the surface charge of the approaching material affects the electric field generated by the proximity sensors. In particular, an approaching hand elicits the largest change in capacitance^41^. The capacitance alternately decreases when a finger periodically approaches within 4 cm of the sensor and decreases further when the distance is reduced to 3 cm, as depicted in the inset of Fig. 4e. More importantly, the position of the approaching finger can be clearly identified, as illustrated by the position contour map in Fig. 4f. Therefore, this soft, capacitor-based sensor array enables the precise location/magnitude identification of pressures loads and approaching objects with high sensitivity, a rapid response, and high reversibility.

### SCMN adjustable sensing range and area expansion

The spatial resolution of human tactile sensing varies across the body. For instance, the spatial resolution at fingertips is about seven times larger than that of palm^56^. As inspired by the variable spatial resolution of mechanoreceptors distributed in different parts of human skin, the SCMN, sensors with multiple sensory functions all integrated in a structured PI network as distributed layout (Supplementary Fig. 11a), does allow the density of the sensory nodes to be adjusted with the meandering structure stretching to represent areas of the skin with highly differing mechanoreceptor density. Besides, different functional sensors could be fabricated on the same sensory node in stack with insulation of PI dielectric thin films, regarded as stacked layout or 3D integration^57,58^ (schematically shown in Supplementary Fig. 11b), which will contribute to high density of mechanosensation electronics. Our fabricated SCMN, actually, is integrated by a combination of the distributed and stacked layouts (Supplementary Fig. 11c). Additionally, design rules of 3D stacked layouts for the multifunctional sensors are presented in Supplementary Table 1.

Figure 4g schematically illustrates the attachment of SCMN to an artificial hand as e-skin both with and without sensing area expansion, exhibiting superior area adjustability and expandability for multi-stimulus sensing (also depicted in Supplementary Fig. 3). As shown in Fig. 4h, spatial pressure mapping can be achieved before and after 300% expansion of the SCMN, indicating that SCMNs can be used to both identify the position of the pressure load and estimate the size of the loading object even with network stretching/expansion. Indeed, the SCMN could realize a 25-fold (or more) expansion of the sensing area, ranging from the original coverage (16 cm^−2^) to the expandable size (400 cm^−2^), shown in Supplementary Fig. 2. Furthermore, this property can be used to define the detection area not only for pressure but also for other external stimuli (e.g., temperature, in-plane strain, proximity, humidity, optical, magnetic field, etc.). These sensory nodes of the SCMN are adjustable and expandable to predefined locations for multifunctional sensing and, through network sensing area expansion, can be used to emulate the different densities of mechanoreceptors in the human skin.

### Simultaneous multiple stimuli sensing

The fabricated SCMN, integration of temperature, in-plane strain, humidity, UV light, magnetic, pressure, and proximity sensors, can in real time record various signals induced from the external environment simultaneously, as well as differentiate each stimulus because of each flexible sensor having good selectivity and discrimination for external stimuli. The sensing ability and orthogonality between sensors in the SCMN are presented in the form of thumbnails in Supplementary Table 2, and the corresponding index is also shown in Supplementary Table 3.

**Fig. 5:**
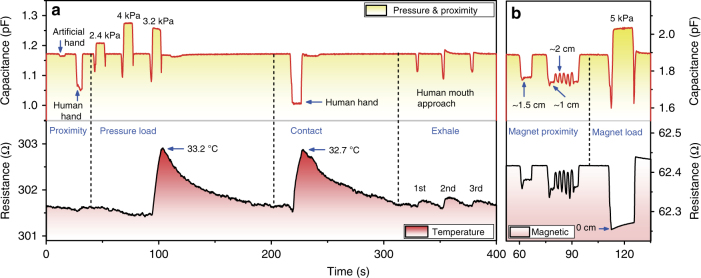
Simultaneous multiple-stimuli sensing performances. **a** Real-time simultaneous sensing of temperature, pressure, and proximity stimuli. Signals in recording: Proximity—artificial/human hand approaches the SCMN with a distance of about 1 cm; Pressure—loading bottles on the SCMN with pressures of 2.4 kPa (empty), 4 kPa (filled with water), and 3.2 kPa (filled with hot water) consecutively; Contact—human hand on the SCMN; and Exhale—human breaths toward the SCMN for three times (Details are described in Supplementary Movie 4). **b** Real-time simultaneous sensing of magnetic field, pressure, and proximity stimuli. The detailed procedures of a permanent magnet put near or loaded on the SCMN: the magnet approaches the SCMN with a distance of about 1.5 cm; the proximity distances change between 1 cm and 2 cm alternatively; the magnet loaded on the SCMN corresponding to a pressure of 5 kPa (see Supplementary Movie 5)

Figure 5a shows real-time recording signals in temperature, pressure, and proximity simultaneously. The procedure involves artificial/human hand proximity, three consecutive pressure loads, human hand contact, and three-time breathing toward the SCMN. When an artificial wood hand or a human hand is brought to approach the SCMN with a distance of about 1 cm, the capacitances of the capacitor-based soft sensor are both triggered to decrease as the interferences of fringe electric field in non-contact mode. It is very noticeable that human hand approaching would lead to a much larger reduction in capacitance. However, the resistance of the temperature sensor does not show any obvious change during proximity sensing. Then placing three bottles (empty, filled with water, filled with hot water) on the SCMN in sequence. On the one hand, the capacitances of the pressure and proximity sensor exhibit corresponding increase rates with applying pressures (2.4, 4, and 3.2 kPa). On the other hand, the resistance of the temperature sensor begins to cause a sharp increase (up to a temperature of 33.2 °C correspondingly) when loading the third bottle that is filled with hot water and then recovers to the range of room temperature after removal of the bottle. With human hand contacts on the SCMN, both signals from proximity and temperature sensing change simultaneously. The capacitance falls to the lowest level, and the resistance arises to a corresponding skin temperature at 32.7 °C. Similarly, human breaths (exhale, containing moisture and heat) toward the SCMN for three times could bring the same change trends in capacitance and resistance as human hand contacts. Additionally, the detailed procedure is also shown in Supplementary Movie 4. Fig. 5b illustrates real-time multiple stimuli sensing as a permanent magnet put near or loaded on the SCMN. The magnetic field from the magnet causes a drop in resistance of the magnetic sensor when the magnet is moving close to the SCMN. Meanwhile, the fringe electric field of the soft sensor disturbed by the magnet also leads to a decrease in capacitance. With the proximity distances change between 1 cm and 2 cm alternatively, the resistance and the capacitance both show periodical fluctuations. Furthermore, a pressure of 5 kPa loaded on the SCMN by introducing the magnet induces an increase in capacitance for pressure sensing and makes it reach the minimum of resistance for magnetic sensing at the same time. The presence of magnet proximity/loading can be seen in Supplementary Movie 5. We can see the SCMN simultaneously monitoring three or more stimuli in Supplementary Fig. 12 as well. Notably, the specific sensor has a good selective detection for its sensitive stimuli, only with a little disturbances induced by other stimuli. More specifically, the coupled process of human finger contact/touch (Fig. 5a, and Supplementary Fig. 12a) and human exhale toward the SCMN (Fig. 5a, Supplementary Figs. 12b, c) can be recorded in real time and differentiated simultaneously.

### Personalized intelligent prosthetic hand with pressure and temperature sensing

**Fig. 6:**
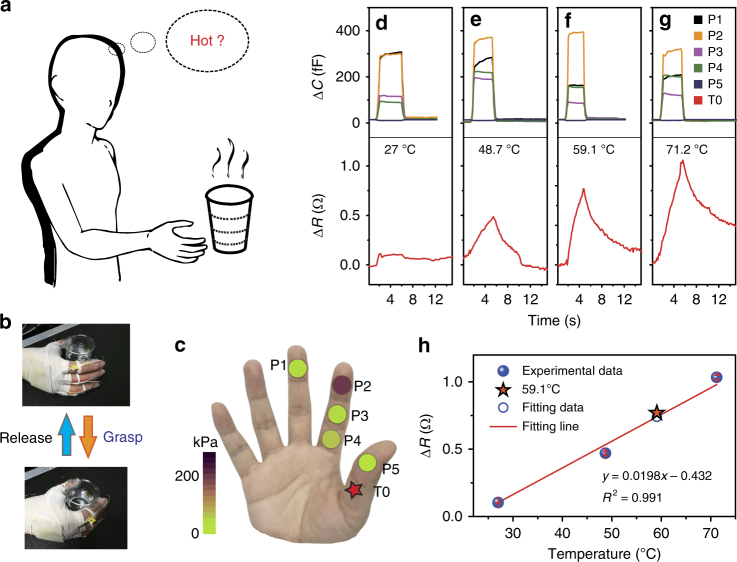
The intelligent prosthetic hand with personalized SCMN configuration. **a** Schematic illustration of human hand picking up a cup with temperature detection at the same time. **b** Images show operations of the intelligent prosthetic hand to grasp (upper) and release (bottom). The procedures of grasping/releasing are also shown in Supplementary Movie 6. The intelligent prosthetic hand equipped with five pressure sensors (P1, P2, P3, P4, and P5) and one temperature sensor (T0) on fingers. **c** Pressure distribution contour on the fingers when the intelligent prosthetic hand is grasping a water cup and sensing temperature of 59.1 °C. **d**–**g** The intelligent prosthetic hand grasps/releases cups filled with water with four different temperatures: 27 °C (**d**), 48 7 °C (**e**), 59.1 °C (**f**), and 71.2 °C (**g**). **h** Temperature estimation by the fitting equation when the intelligent prosthetic hand is grasping

These capabilities of sensing pressure and temperature are essential features of the human skin^3,4^. As schematically shown in Fig. 6a, human hand can naturally differentiate temperatures when touching. Hence, we construct an intelligent prosthetic hand in personalized SCMN configuration, consisting of pressure and temperature sensor pixels on fingers, to facilitate simultaneous and sensitive pressure and temperature sensing. Pressure sensors integrated into the prosthetics could help disabled patients to regain the functionality of force sensing for grasp control and object manipulation. Furthermore, sensing temperature can provide information about the surroundings and avoid damaging temperatures. When the prosthetic hand is going to grasp a cup filled with water, temperature sensor would achieve cold/hot sensation for water and contribute to prevent injury from high temperatures. Notably, the prosthetic hand, adding the intelligent factor by means of multiple sensors integration, can not only contour pressure distribution on fingers but also estimate temperatures of grasping objects simultaneously.

The grasping and releasing operations of the intelligent prosthetic hand are visually described in Fig. 6b and Supplementary Movie 6. The intelligent prosthetic hand equipped with five pressure sensors (P1, P2, P3, P4, and P5) and one temperature sensor (T0) on fingers. Herein P1 is on the middle finger, P2, P3, and P4 are on the forefinger, and P5 and T0 are on the thumb, respectively. Their positions are also presented in Fig. 6c. By optimizing testing circuitry and shield setting, the interference from proximity signals is minimized, leading to a better sensing performance in contact mode. The intelligent prosthetic hand grasps/releases cups filled water with four different temperatures (water temperatures are measured with an infrared thermometer, respectively) are illustrated in Fig. 6d–g. When the intelligent prosthetic hand touches the cup, five pressure sensors (P1, P2, P3, P4, and P5) on fingers begin to increase the capacitance in pace with the increase of grip strength quickly, then the capacitance keeps small variations over a period of time at the peaks of grip strength. Meanwhile, temperature sensor (T0) also exhibits increment trend of resistance change at various degrees according to different water temperatures. The higher temperature causes a larger resistance change, which is just the same as mentioned above. Furthermore, capacitances of all five pressure sensors fast recover to the original values, and resistances of temperature sensor show recovery behavior as well, when the intelligent prosthetic hand coming to release. Impressively, pressure distribution on the fingers is illustrated in Fig. 6c, while the intelligent prosthetic hand grasping a cup with water temperature of 59.1 °C is illustrated in Fig. 6f. Compared with other pressure sensors on the fingers, the pressure sensor (P2) exhibits the largest capacitance change, which means that P2 bears the maximum grip pressure (about 200 kPa) in the grasp operation. Moreover, we are able to derive the linear relation between temperature and resistance according to the resistance values from 27, 48.7, and 71.2 °C. As shown in Fig. 6h, the resistance value from 59.1 °C is very close to the fitting one, representing a small variation of 2.6%. In effect, we can do temperature estimation through the experimental data and the fitting equation when the intelligent prosthetic hand is performing grasp/release operations. Briefly, the personalized intelligent prosthetic devices could allow amputees or individuals with nerve damage to regain considerable functionalities, such as touch and temperature sensing, to improve the rehabilitation and transform the lives and abilities of the disabled patients.

## Discussion

Skin-inspired highly stretchable and conformable matrix networks have been fabricated that integrate temperature, strain, humidity, light, magnetic, pressure, and proximity sensors as multifunctional detection matrix. The SCMN presented in this work successfully expands the sensing functionality of e-skin into seven categories, and it can simultaneously detect and differentiate three or more stimuli. Take the magnetic sensing and the pressure sensing as examples (Fig. 6b, Supplementary Movie 5, Supplementary Figs. 9 and 10), the SCMN works very well in different environment, like changing temperature, RH, UV intensity, or in-plane strain. Moreover, the sensing area of the SCMN can be adjusted and predefined by stretching and expanding the meandering interconnects, which acted as neural pathways. This skin-inspired SCMN, which features simultaneous multi-stimulus sensing, adjustable sensing range/large-area expandability, and potential high-density 3D integration, can sense the magnitude and location of multi-stimulus sensing in real time, impressively construct a personalized intelligent prosthesis for pressure/temperature sensing, and will likely attract considerable attention for its application to humanoid robotics, new prosthetics, human–machine interfaces, and health-monitoring technologies.

## Methods

### Fabrication of the highly stretchable and conformable PI networks

First, the Kapton HN film (DuPont) was cleaned by ultra-sonication with acetone, alcohol, and deionized water for 10 min; dried by N_2_ flow; and cured in an oven at 80 °C for 4 h. Second, a 3-inch silicon wafer was spin-coated with a 4-μm-thick photoresist layer, and then the Kapton film was bonded to the wafer using the hot-press method. Third, after being spin-coated with a 2-μm-thick negative photoresist layer (NR9-1500PY), the Kapton film was patterned by UV lithography (Suss MicroTech, MA 6). Fourth, Au (or Cr/Cu/Al/Ni) thin film (DC 100 W, 3 mTorr, 10 min) was deposited by sputter coating (Denton Vacuum, Discovery 635) and then lifted off to form a patterned Au mask. Fifth, the patterned Kapton film was etched (O_2_ plasma, 150 mTorr, 100 W) using a reactive ion etcher (South Bay Tech., RIE 2000). Finally, the etched Kapton film was released, and the metal mask was removed by wet or dry etching to obtain the highly stretchable and conformable PI networks.

### Fabrication of expandable sensors and soft sensor array integrated on the SCMN

The fabrication process is illustrated in Supplementary Fig. 1. Step 1: A layer of poly (methyl methacrylate) (PMMA; 4,000 r.p.m. for 60 s, baked at 150 °C for 30 min) and a layer of PI (polyamide acid solution; 2,000 r.p.m. for 60 s and 4 h at 250 °C, four times to form a 30-µm film) were sequentially spin-coated onto a Si wafer. Step 2: Metal electrodes and dielectric layer preparation. Photolithography (NR9-1500PY, 2 µm), sputtered aluminum thin films, and lift-off photoresist were used to pattern the row and column meandering structures, and a PI dielectric layer (500 nm) was prepared between the electrodes. Step 3: Expandable sensor preparation consisted of photolithography (NR9-1500PY, 2 µm) to generate the sensor patterns, sputtering of the specific sensitive elements (Pt for temperature, Constantan for in-plane strain, Co/Cu multilayer for magnetic field, Al/PI for humidity, Al/ZnO for UV light, etc.), lift-off of the photoresists, and encapsulation of the patterned PI layer (3 µm). Additionally, the specific sensor patterns and elements for each stimulus are available in the literatures^30,31,52,53,59–61^. Step 4: Network preparation consisted of photolithography (NR9-1500PY, 2 µm) followed by sputtering of a SiO_2_ etching mask, lift-off of the photoresist, and reactive ion etching (RIE, O_2_ plasma, 150 mTorr, 200 W). Step 5: Network pickup and soft sensor array preparation. Immersion in hot acetone partially removed the PMMA layer, and the network was retrieved from the silicon wafer and attached to a piece of poly(vinyl alcohol) (PVA) or poly(dimethylsiloxane) (PDMS). Sputtered Ag thin films formed the bottom and top electrodes via a mask on the opposite side of the etched network structure, and Ecoflex silicone elastomers (Smooth-On 0010, mixing part A and part B with the ratio of 1:1 in weight, 300 r.p.m. for 50 s, cured at room temperature over one night, and cut into pad size) were placed on the sensory nodes between the two electrodes. Finally, some certain amounts of PDMS or PVA solutions were poured uniformly on the fabricated network and then baked at 60 °C for several hours. The SCMN with a layer of PDMS or PVA can be obtained.

### Characterization of the integrated sensors

SEM images were obtained on a Hitachi SU8020. Applied force was measured using a force gauge (ATI, Nano 17). Sensor resistance and capacitance measurements were recorded on an Agilent (E4980A) Precision LCR meter with custom LabVIEW programs, and sensor currents were measured on a Keithley 4200 SC.

### Data availability

All data supporting this study and its findings are available within the article and its Supplementary Information or from the corresponding author upon reasonable request.

## Electronic supplementary material

Supplementary Information

Description of Additional Supplementary Files

Supplementary Movie 1

Supplementary Movie 2

Supplementary Movie 3

Supplementary Movie 4

Supplementary Movie 5

Supplementary Movie 6
