# Supplementary material for: Skin-inspired highly stretchable and conformable matrix networks for multifunctional sensing
Source: Nat Commun. 2018 Jan 16;9:244. doi: 10.1038/s41467-017-02685-9 (PMC5770430; doi:10.1038/s41467-017-02685-9)
Supplement: Supplementary file 1 — Supplementary Information [file 41467_2017_2685_MOESM1_ESM.pdf]

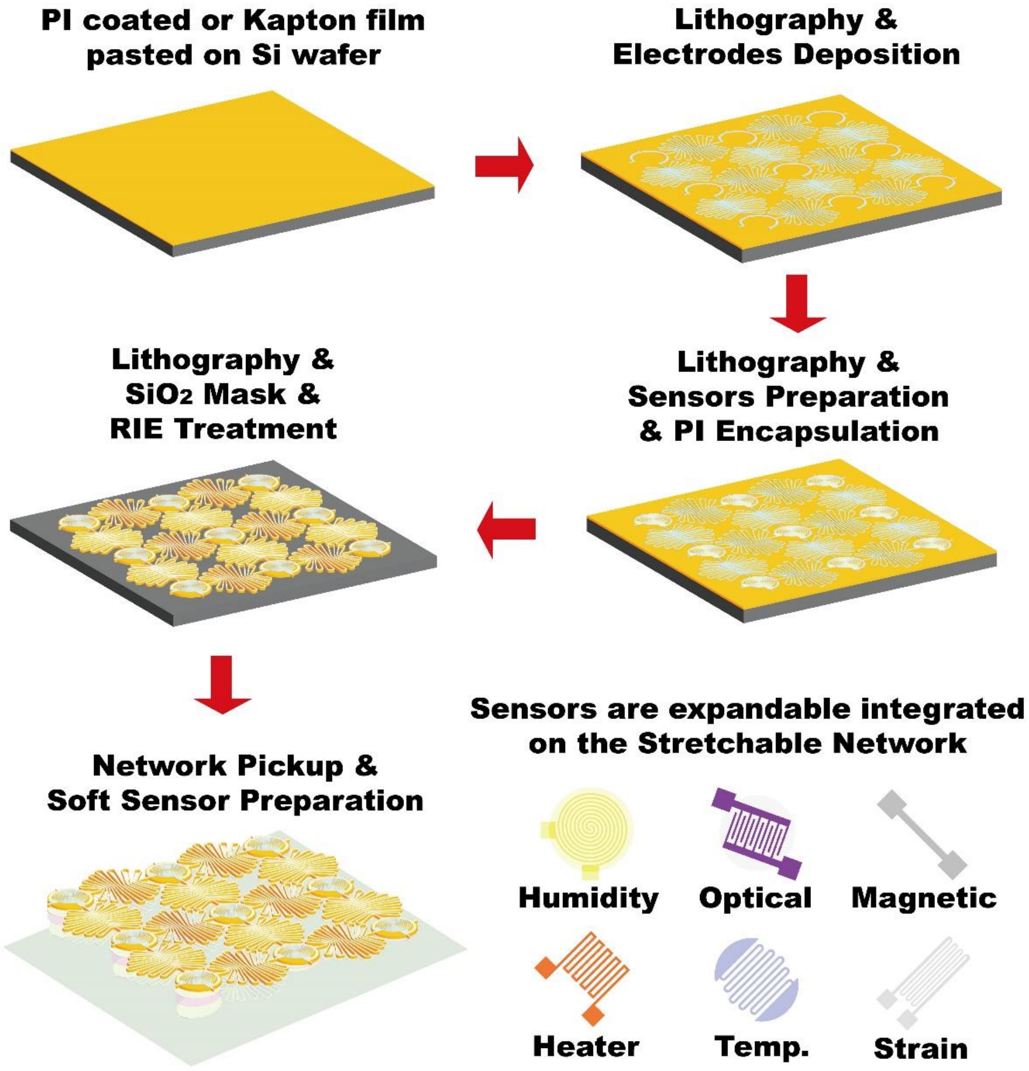

Supplementary Figure 1 | Schematic overview of SCMN fabrication.

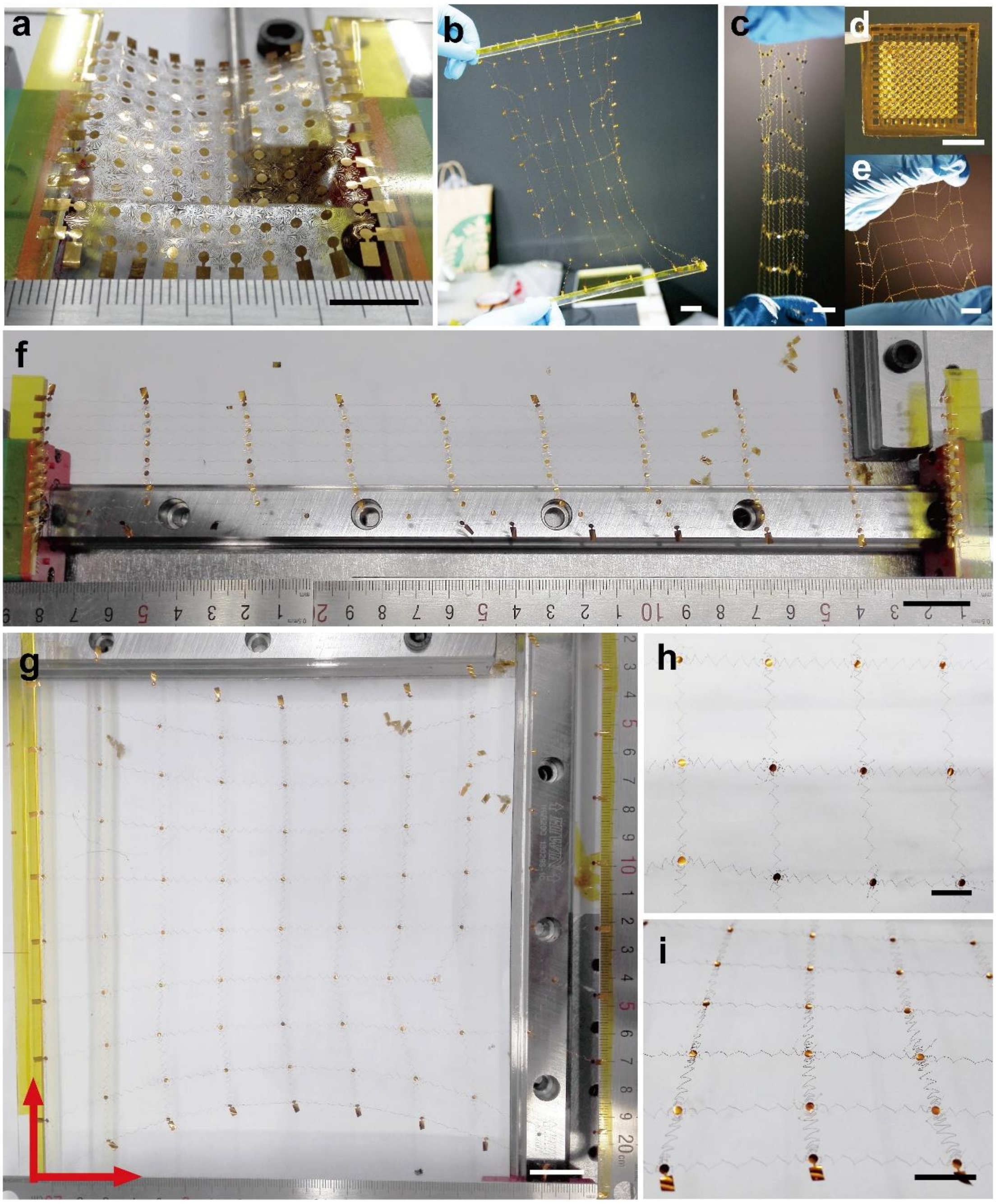

Supplementary Figure 2| Evaluation of stretchability and expandability. (**a**) Before the network is stretched. (**b**) The network is stretched by hand (scale bar: 2 cm). (**c-e**) Images of a small network before and after stretching and expansion (scale bar: 1 cm). (**f**) Network expansion by 675% along the x-axis. (**g**) Image of 2500% SCMN expansion (scale bar: 2 cm), realizing a 25-fold expansion of the sensing area (400 cm^{-2}) from its original state (16 cm^{-2}). (**h, i**) Enlarged view of (f) (scale bar: 1 cm).

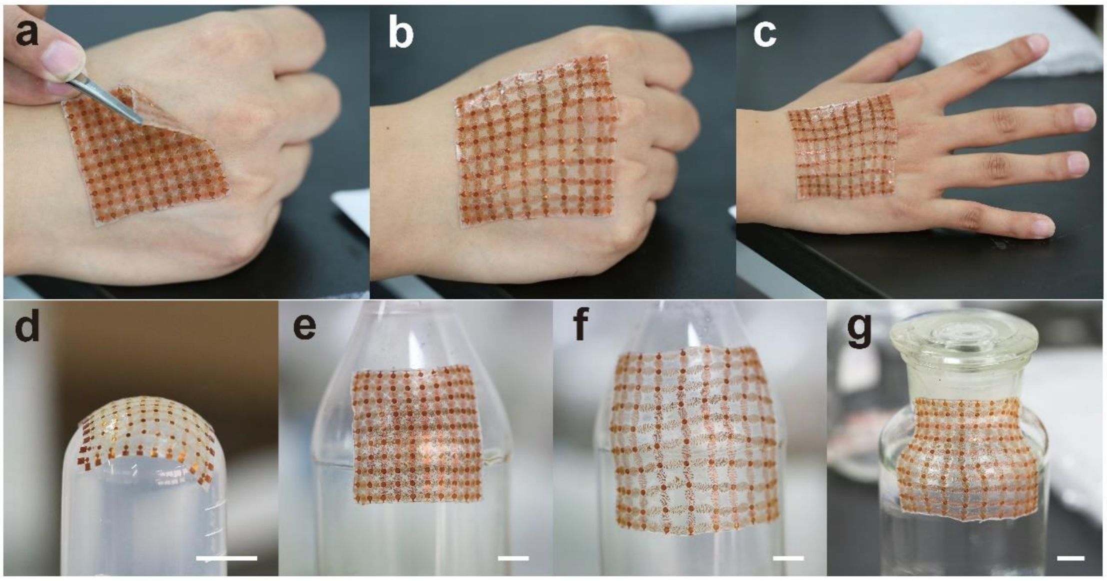

Supplementary Figure 3 | A thin, compliant, skin-like SCMN. (**a-c**) An SCMN conforming to a human hand. (**d**) An SCMN attached to a dome. (**e-g**) SCMNs attached well to plastic or glass bottles with various curvatures; size expansion of an SCMN is shown in (**f**). (scale bar: 1 cm).

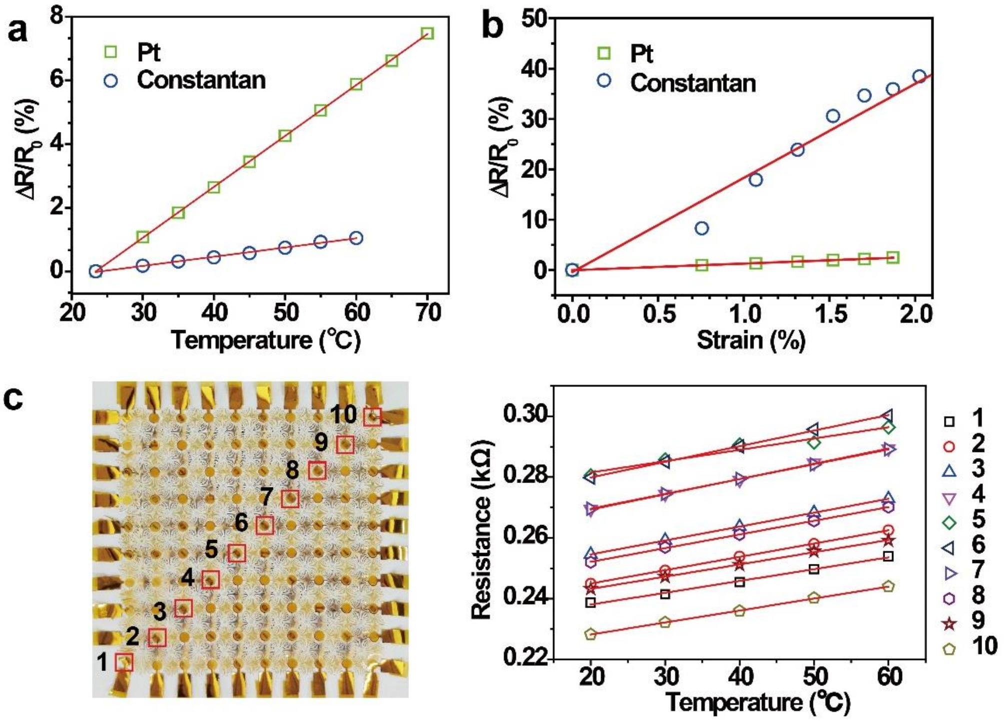

Supplementary Figure 4 | Temperature and in-plane strain sensing. (**a**) Relative resistance changes for Pt and Constantan alloy induced by temperature. (**b**) Relative resistance changes for Pt and Constantan alloy induced by strain. (**c**) Ten temperature sensors fabricated diagonally on an SCMN (left), and linear changes in sensor resistance with temperature (right).

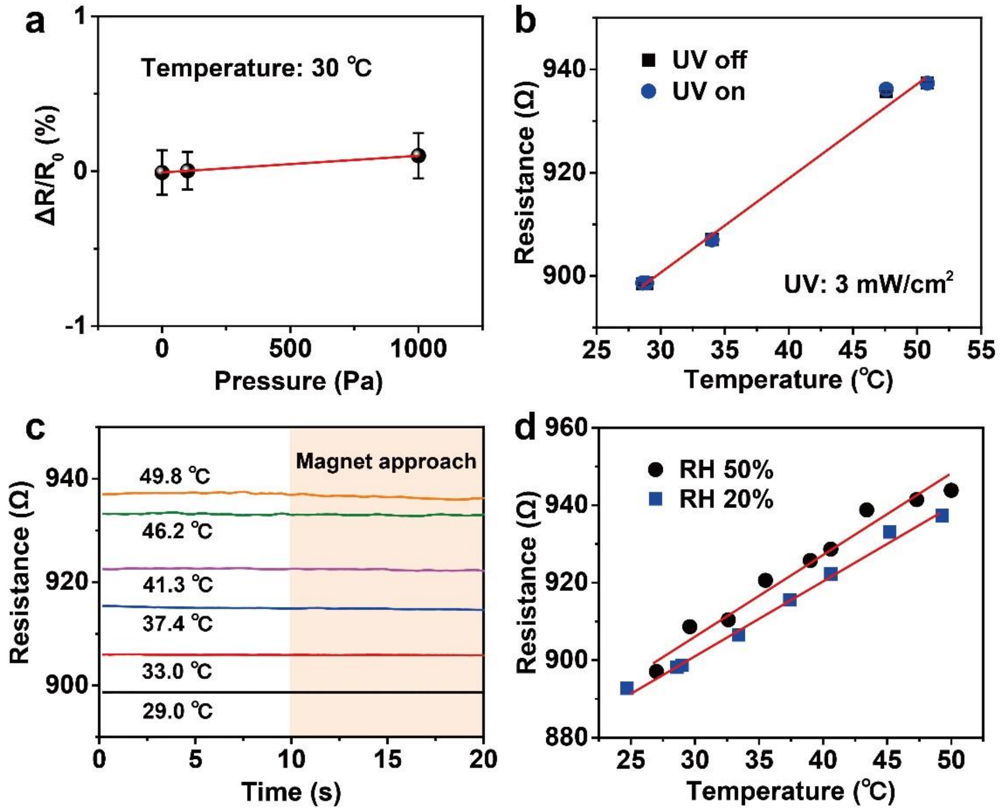

Supplementary Figure 5 | External stimulus responses of the temperature sensors. (**a-d**) Resistance responses of the temperature sensors under pressure, UV light, magnetic field and RH. Error bars in (a) indicate the standard deviations calculated from 10-time cyclic pressure loading tests.

Temperatures differentiate from other external stimuli are presented here. Relative resistance change in the temperature sensor has a very small growth (~0.1%) as applied pressure up to 1000 Pa, shown in Supplementary Fig. 5a. Changes in resistance both have no strong variations at different temperatures when UV light (3 mW/cm^2) turns on-off state (Supplementary Fig. 5b) or a permanent magnet approaches (Supplementary Fig. 5c). Supplementary Figure 5d demonstrates that resistances of 50% RH are little bit higher than that of 20% RH when changing temperature, but the two linear correlations between resistance and temperature are quite comparable. As has been stated above, it surely shows a favorable selectivity in temperature for the SCMN.

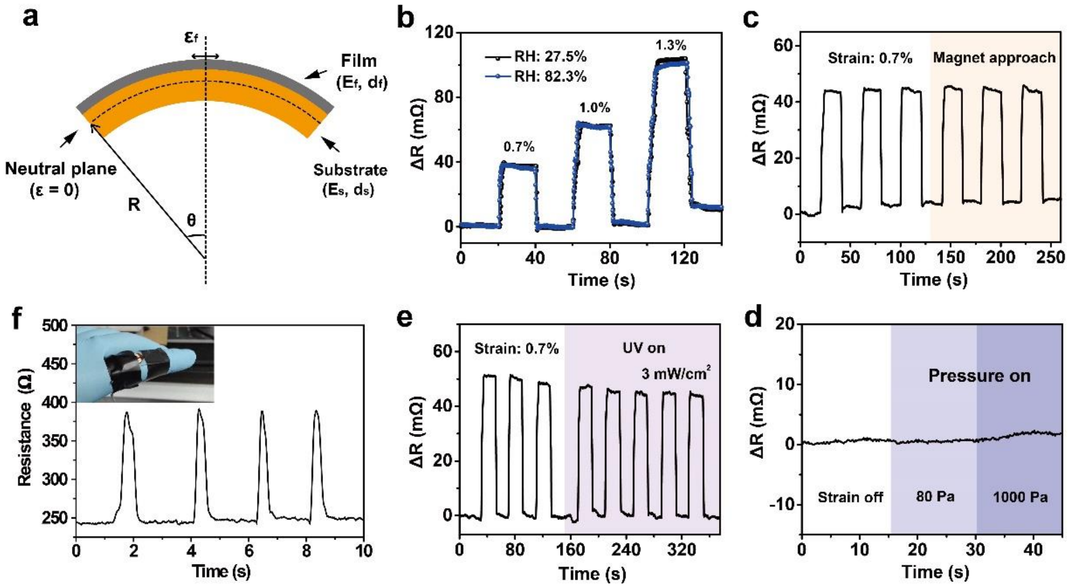

Supplementary Figure 6 | External stimulus responses of the in-plane strain sensors. (**a**) Schematic cross-section of the mechanical model used for in-plane strain calculations. (**b-d**) Resistance responses of the strain sensors under RH, magnetic field, pressure and UV light. (**f**) Strain recording for finger bending manner.

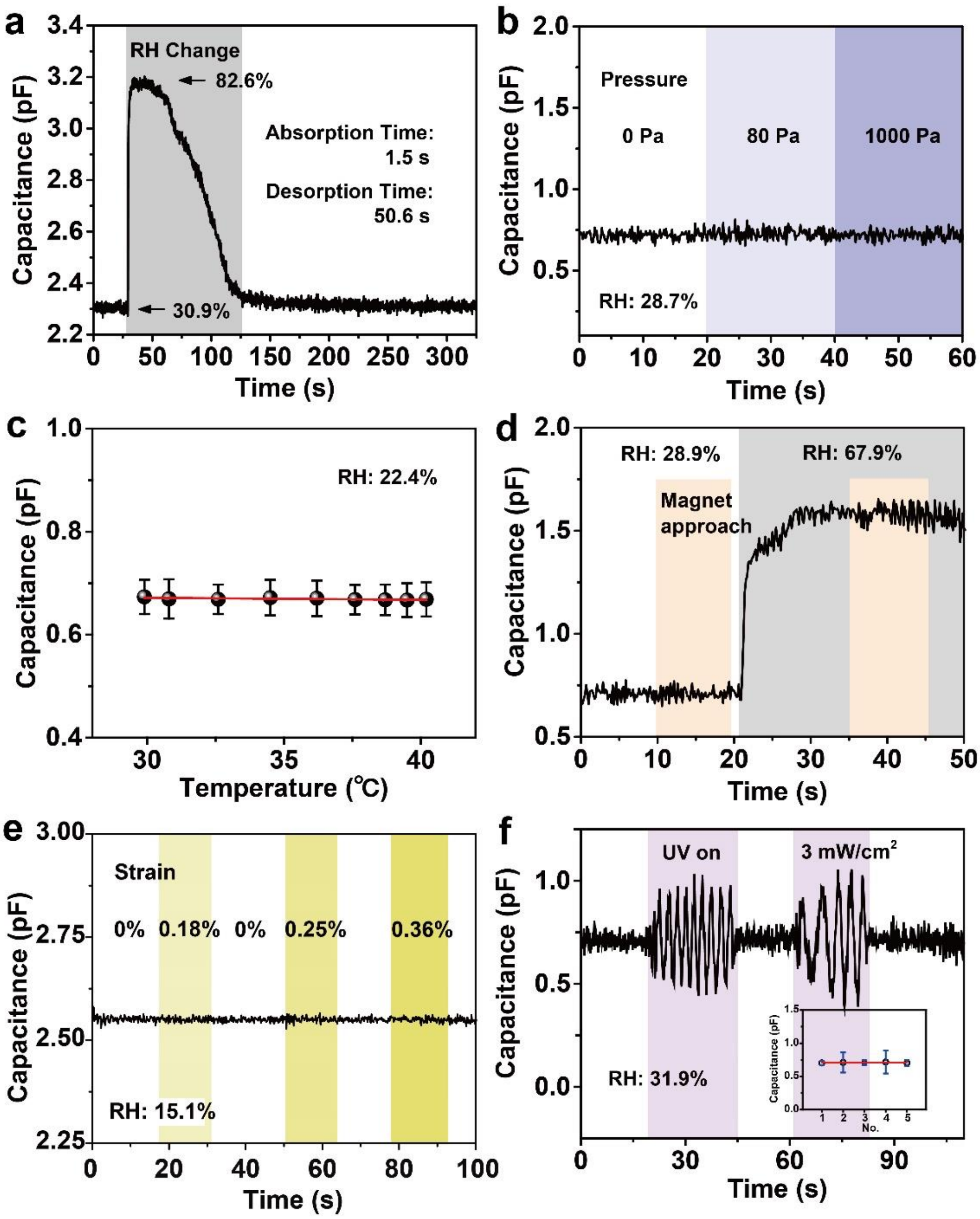

Supplementary Figure 7 | External stimulus responses of the humidity sensors. (**a**) Temporal capacitance change in the humidity sensor. (**b-f**) Capacitance responses of the humidity sensors under pressure, temperature, magnetic field, in-plane strain and UV light. The inset of (f) shows the variations in capacitance when UV light is on or off. Error bars in (c,f) represent the fluctuation of capacitance at each point under temperature and UV light, respectively.

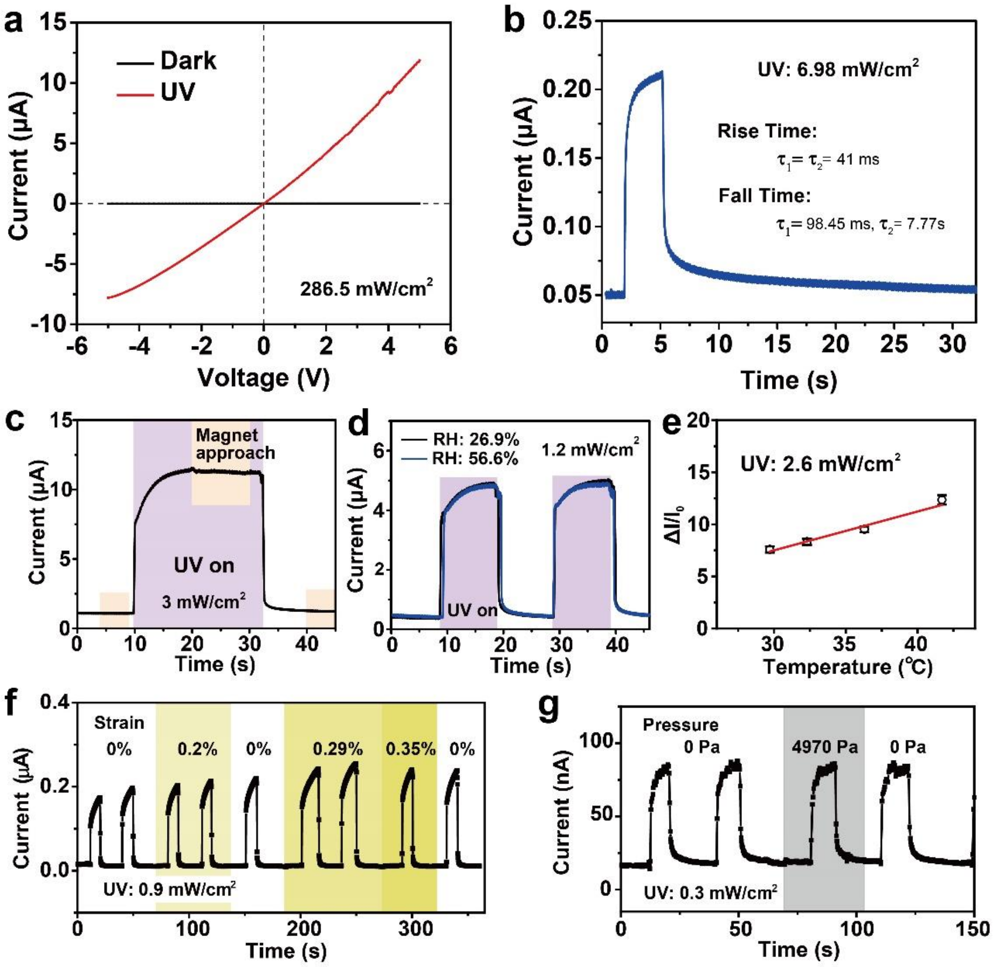

Supplementary Figure 8 | External stimulus responses of the UV light sensors. (a) I–V characteristics of the optical sensor in the dark and under 355 nm UV illumination (286.5 mW/cm^2) at a 5 V bias. (**b**) Temporal response of the ZnO UV detector. (**c-g**) Current responses of the ZnO UV detector under magnetic field, RH, temperature, strain and pressure, respectively. Error bars in (e) represent the fluctuation between normalized current and temperature at each point.

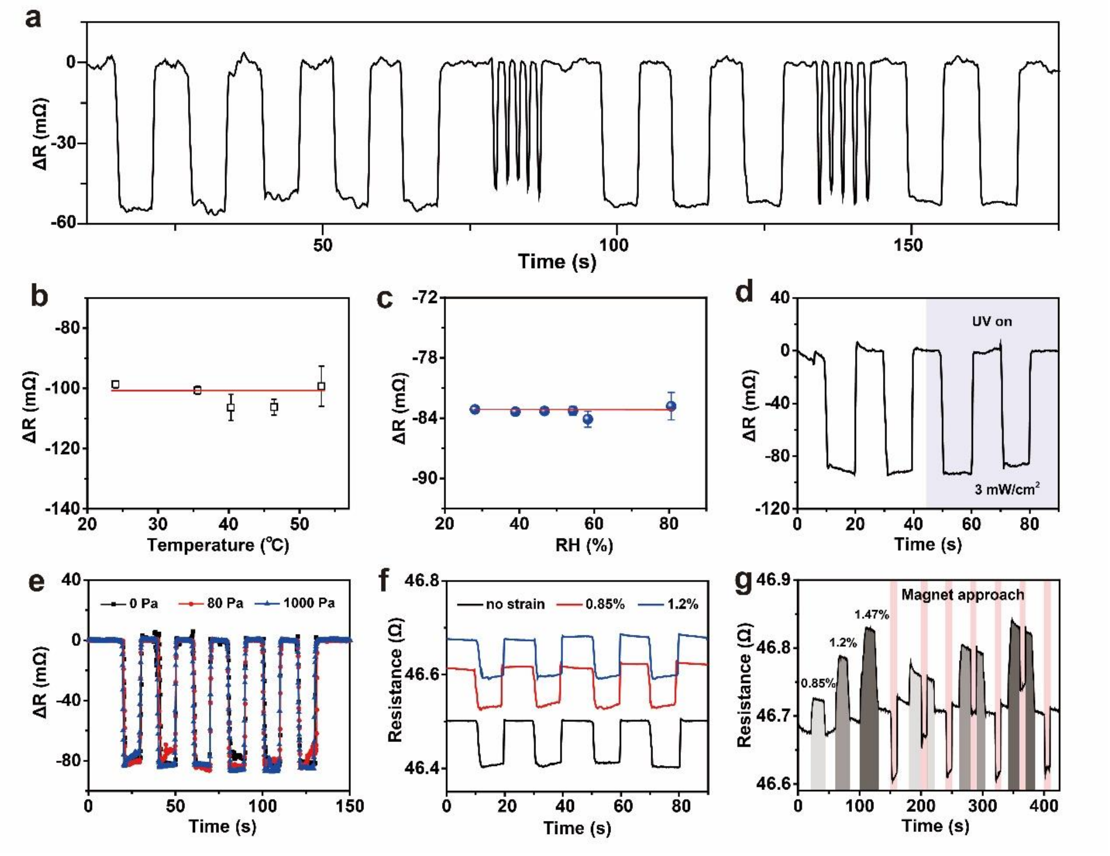

Supplementary Figure 9 | External stimulus responses of the magnetic sensors. (**a**) Resistance changes recorded during cyclical and repeated approach a magnet to or withdrawal from the sensor, covering the full range of Fig. 3j. (**b**-**f**) The changes in resistance induced by the approach of a magnet do not vary with temperature (b), RH (c), UV illumination (d), pressure (e) or in-plane strain (f). (**g**) Resistance changes recorded during the application of three different strains (0.85%, 1.2% and 1.47%) independently and in conjunction with an approaching magnet (pink). Error bars in (b,c) represent the fluctuation of resistance change at each point under temperature and RH, respectively.

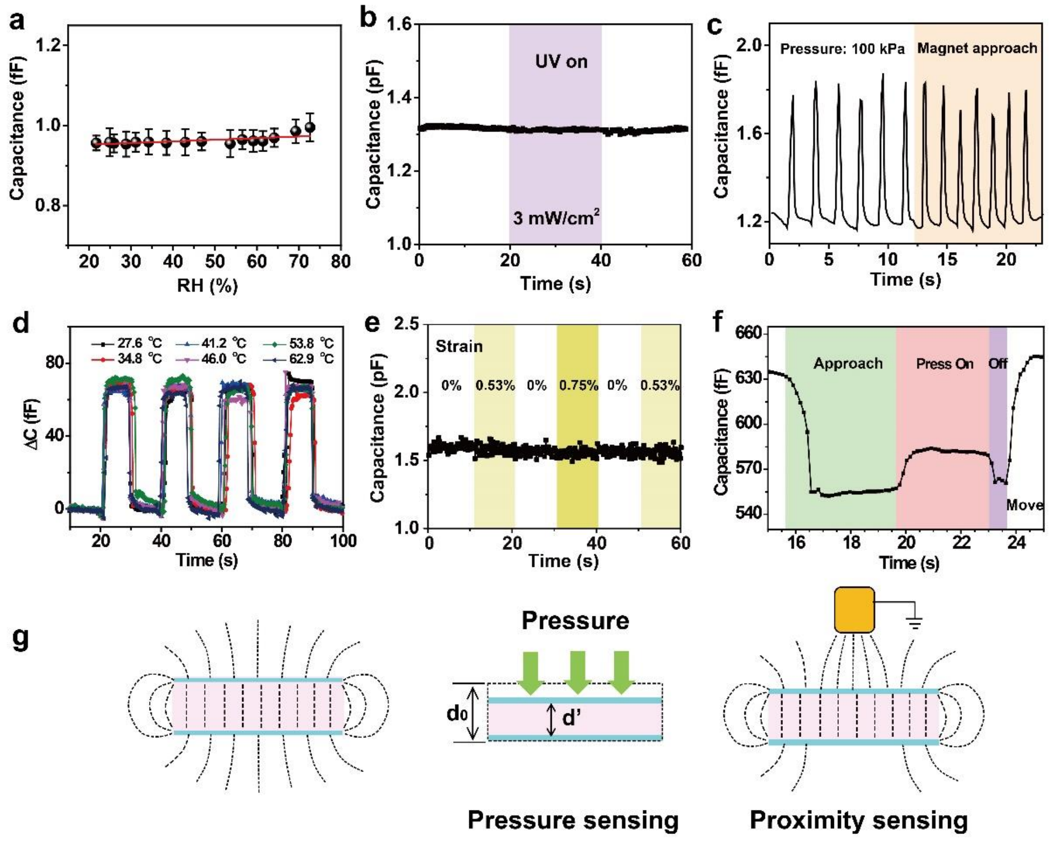

Supplementary Figure 10 | Pressure and proximity sensing. (**a**) The relationship between capacitance and RH (Error bars represent the fluctuation of capacitance at each point under RH). (**b**) Capacitance shows no obvious change by UV illumination (3 mW/cm^2). (**c**) Pressure detection with cyclical 100 kPa loading is not affected by magnetic field. (**d**) The variations of capacitance change are observed to be similar with increasing temperature. (**e**) The capacitance responses to the applied strains. (**f**) Procedure analysis of capacitance changes while proximity and pressure modes both are present in Fig. 4d. (**g**) Schematic illustration of the pressure and proximity sensing mechanism.

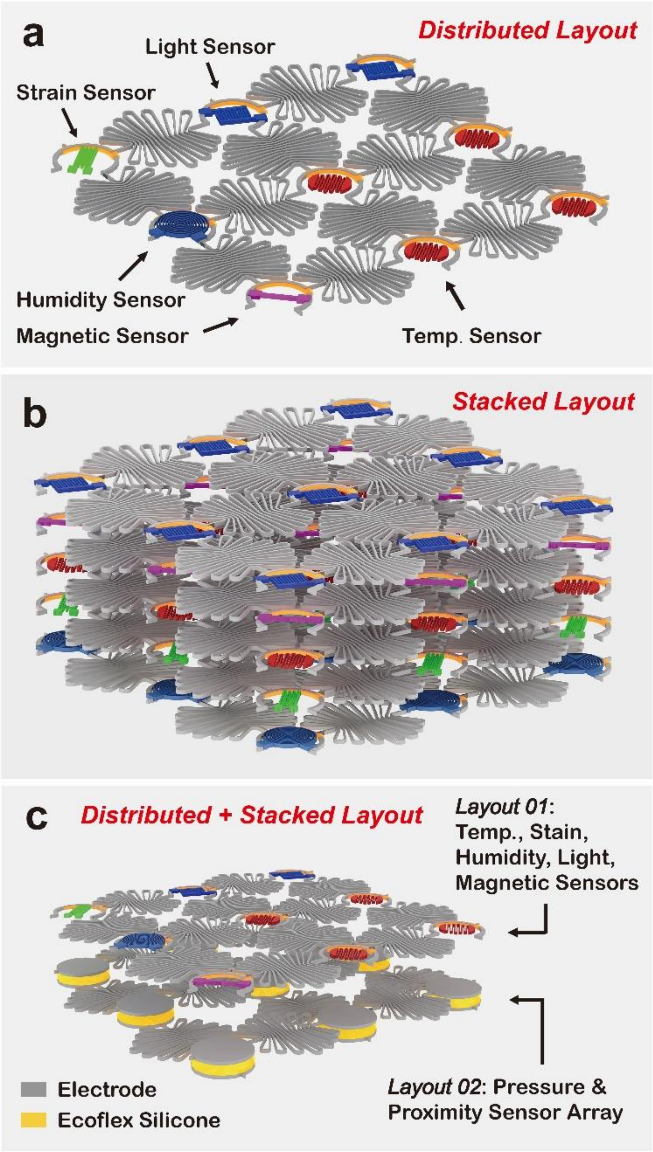

Supplementary Figure 11 | Schematic illustrations on integration schemes of multifunctional sensors in the SCMN. The PI substrates were not shown here to clearly see different layers of sensors. (**a**) Distributed Layout - Sensors with multiple sensory functions (e.g., light, strain, humidity, magnetic, temperature, etc.) all integrated on a structured polyimide network. (**b**) Stacked Layout - Sensors with multiple sensory functions (e.g., humidity, strain, temperature, magnetic, light, etc.) fabricated on the same sensory node in stack, also regarded as 3D integration for mechanosensation electronics. (**c**) Distributed and Stacked Layout for our SCMN device integration. Layout 01 includes temperature, strain, humidity, light and magnetic sensors (or arrays). Layout 02 is the capacitor-based sensors (pressure and proximity sensing).

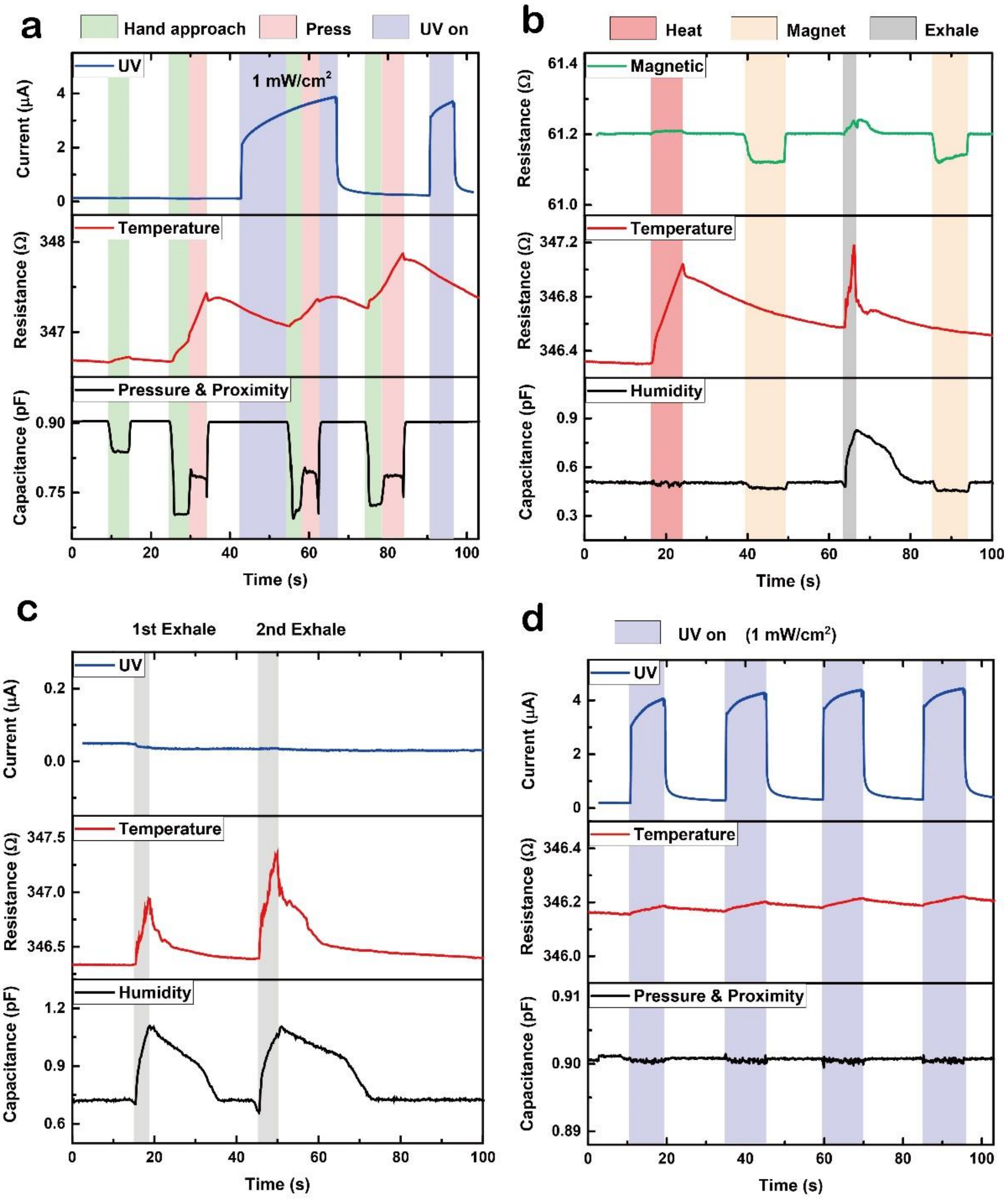

Supplementary Figure 12 | Simultaneous multiple-stimuli sensing performances. (**a**) Real-time simultaneous sensing of UV light, temperature, pressure and proximity stimuli. Hand proximity (green), load pressure (pink), and UV light on (1 mW/cm^2, blue). (**b**) Real-time simultaneous sensing of magnetic, temperature, and humidity stimuli. A heat source approaching (red), a magnet proximity (orange), and human exhale towards SCMN (gray). (**c**) Real-time simultaneous recording signals of UV light, temperature and humidity sensors for two-time human exhale towards the SCMN. (**d**) Real-time simultaneous recording signals of UV, temperature, and pressure and proximity sensors in the SCMN responding to UV light on/off.

Supplementary Table 1 | Design rules of 3D stacked layouts for the multifunctional sensors.

Layout	Integration	Sensors	Design Rules
1	not stackable	Humidity sensor	✓ Need to interact with the ambient directly due to the sensing mechanism, those sensors should be at the outmost layout with a distributed integration.<br>✓ Compatible fabrication process
		Light sensor	
2	stackable	Temp. sensor	
3		Magnetic sensor	
4		Strain sensor	
5		Pressure and proximity sensor	

Supplementary Table 2 | Summary table of sensing ability and orthogonality between sensors in the SCMN.

Sensors \ Sensing	Temperature	Strain	RH	UV	Magnetic field	Pressure
Temperature Sensor	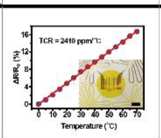	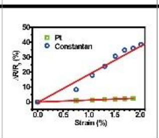	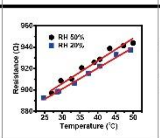	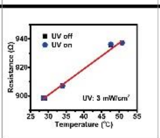	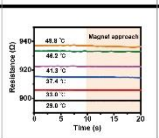	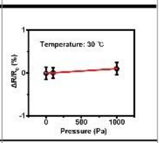
Strain Sensor	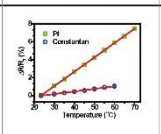	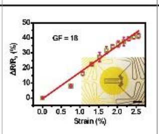	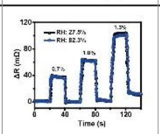	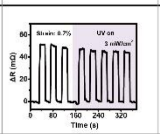	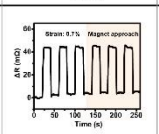	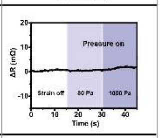
Humidity Sensor	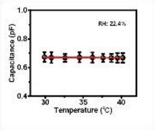	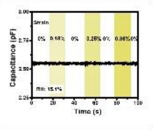	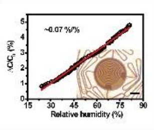	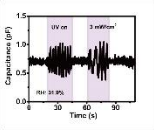	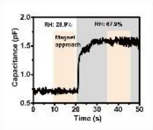	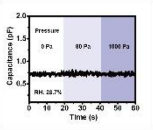
Light Sensor	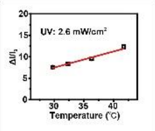	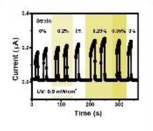	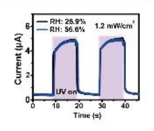	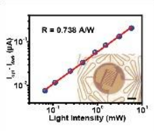	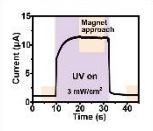	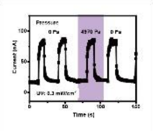
Magnetic Sensor	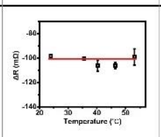	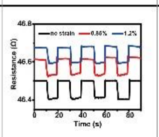	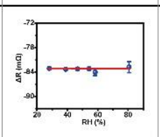	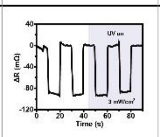	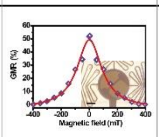	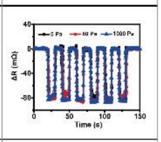
Pressure Sensor	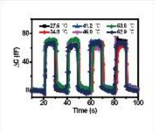	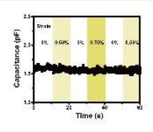	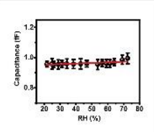	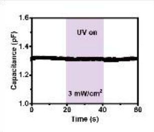	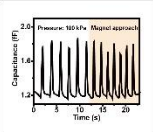	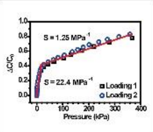

Supplementary Table 3 | The corresponding index in the Supplementary Figures for the orthogonality between the sensors in the SCMN.

	A	B	C	D	E	F
**A (Temp.)**	**X**	**4b**	**5d**	**5b**	**5c**	**5a**
**B (Strain)**	**4a**	**X**	**6b**	**6e**	**6c**	**6d**
**C (RH)**	**7c**	**7e**	**X**	**7f**	**7d**	**7b**
**D (UV)**	**8e**	**8f**	**8d**	**X**	**8c**	**8g**
**E (GMR)**	**9b**	**9f**	**9c**	**9d**	**X**	**9e**
**F (Pressure)**	**10d**	**10e**	**10a**	**10b**	**10c**	**X**

Supplementary Note 1

For each radius of curvature, the in-plane strain ε_f can be calculated using a bi-layer model with respect to the Young modulus E and thickness values [1, 2].

$$\varepsilon_f = \left(\frac{1}{R} \pm \frac{1}{R_0}\right)\left(\frac{d_s + d_f}{2}\right)\frac{1+2\eta+\chi\eta^2}{(1+\eta)(1+\chi\eta)} \qquad \text{Equation (1)}$$

where R and R_0 are the applied and initial radii of curvature, respectively, d_f and d_s are the thicknesses of film and substrate, respectively, η is defined by $\eta = d_f/d_s$, and χ is defined by $\chi = E_f/E_s$, where E_f and E_s are the Young moduli of the film and substrate, respectively. The plus (or minus) sign depends on applied bending opposite to (or with) the built-in curvature.

For $d_f \ll d_s$, Equation (1) can be simplified as:

$$\varepsilon_f = \pm \frac{d_s}{2R} \qquad \text{Equation (2)}$$

where the plus (or minus) sign represents tensile (or compressive) bending strain. Moreover, when the film/substrate is bent at a radius of curvature R, the corresponding chord length, central angle and arc length are C, θ, and L, respectively. The relationships can thus be expressed as: $2\theta \cdot R = L$ and $\sin\theta = C/(2 \cdot R)$. Here, the chord length C can be obtained as a function of the radius, expressed as:

$$C = 2R \cdot \sin(L/2R) \qquad \text{Equation (3)}$$

Therefore, the bending radius R can be determined by controlling the values of the chord length C. Finally, we obtain the corresponding strain ε_f of the film at each radius of curvature.

Supplementary Reference

1 Gleskova, H., Wagner, S. & Suo, Z. Failure resistance of amorphous silicon transistors under extreme in-plane strain. *Appl. Phys. Lett.* **75**, 3011-3013 (1999).

2 Kervran, Y. *et al.* Microcrystalline silicon: strain gauge and sensor arrays on flexible substrate for the measurement of high deformations. *Sens. Actuators A-Phys.* **236**, 273-280 (2015).
