# Supplementary material for: Skin-inspired highly stretchable and conformable matrix networks for multifunctional sensing
Source: Nat Commun. 2018 Jan 16;9:244. doi: 10.1038/s41467-017-02685-9 (PMC5770430; doi:10.1038/s41467-017-02685-9)
Supplement: Supplementary file 2 — Description of Additional Supplementary Files [file 41467_2017_2685_MOESM2_ESM.pdf]

Description of Additional Supplementary Files

File Name: Supplementary Movie 1
Description: A meandering wire is stretched up to 700% expansion ($L/L_0 = 7$).

File Name: Supplementary Movie 2
Description: A small polyimide network is stretched and expanded by human hand along x-axis direction.

File Name: Supplementary Movie 3
Description: A pre-stretching polyimide network along y-axis direction achieves the 2500% expansion of the sensing area. (Speed ×2.5)

File Name: Supplementary Movie 4
Description: Real-time simultaneous sensing of pressure, proximity and temperature. Signals in recording: Proximity - artificial/human hand approaches the SCMN with a distance of about 1 cm; Pressure - loading bottles on the SCMN with pressures of 2.4 kPa (empty), 4 kPa (filled with water) and 3.2 kPa (filled with hot water) consecutively; Contact - human hand on the SCMN; and Exhale - human breathes towards the SCMN for three times. (Video recording for testing as Fig. 5a)

File Name: Supplementary Movie 5
Description: Real-time simultaneous sensing of pressure, proximity and magnetic field. Signals in recording: pressure loading by putting bottles (2.4 kPa and 4 kPa) on the SCMN, respectively; the magnet approaches the SCMN with a distance of about 1.5 cm; the proximity distances change between 1 cm and 2 cm alternatively; the magnet loaded on the SCMN corresponding to a pressure of 5 kPa. (Video recording for testing partially as Fig. 5b)

File Name: Supplementary Movie 6
Description: The grasping and releasing operation process of the intelligent prosthetic hand with personalized SCMN configuration.
